# Characterization of the mitochondrial genome of *Alloxiphidiopsis emarginata* (Orthoptera, Tettigoniidae, Meconematinae)

**DOI:** 10.1080/23802359.2019.1693288

**Published:** 2019-11-21

**Authors:** Shao Li Mao, Yuan Lu, Lu Lu Xun, Ya Fu Zhou

**Affiliations:** a Xi’an Botanical Garden of Shaanxi Province/Institute of Botany of Shaanxi Province, Xi’an, Shaanxi, China;; b Shaanxi Engineering Research Centre for Conservation and Utilization of Botanical Resources, Xi’an, Shaanxi, China

**Keywords:** *Alloxiphidiopsis emarginata*, meconematinae, mitochondrial genome, phylogeny

## Abstract

The length of *Alloxiphidiopsis emarginata* complete mitogenome was 16,207 bp and contained the typical gene arrangement, base composition, codon usage found in other related species. The overall base composition exhibited obvious anti-G (10.6%) and AT bias (71.6%). The initiation codons of all PCGs were typical ATN (ATA/ATG/ATT), and the termination codons were TAA, TAG, or incomplete stop codon T. All tRNAs could be folded into typical cloverleaf secondary structures, except tRNA^Ser^ (AGN). Phylogenetic analyses showed that *A*. *emarginata* was closer with *Xizicus howardi*.

Meconematinae is a diverse subfamily in Tettigoniidae with more than 42 genera 220 species distributed in China (Cigliano et al. 2019). Despite the high number of species in Meconematinae, only eight species belonged to four genera had been sequenced (Yang et al. 2012; Liu 2017; Zhou et al. 2017; Mao, Qiu et al. 2018; Mao, Yuan, et al. 2018; Han et al. 2019). The genus *Alloxiphidiopsis* was proposed mostly based on the highly modified ninth abdominal tergite of male (Liu and Zhang 2007). The nominate species *A. emarginata* were sequenced by Illumina Hiseq 2500 platform and assembled using MitoZ (Meng et al. 2019). The whole mitochondrial genome sequence was annotated using the software Geneious v 11.1.5 (Kearse et al. 2012). The specimen was collected from Henan province (33°17′N, 110°27′E), China in 2009 and was deposited in herbarium of Xi’an Botanical Garden of Shaanxi Province (no. 19013).

The complete mitogenome of *A. emarginata* is 16,207 bp in length and has been deposited in GenBank (Accession no. MN562488). It consists of 13 protein-coding genes, 22 tRNA genes, 2 rRNA genes, and 1 control region, its structure and arrangement are identical with hypothesized ancestral insect mitogenome (Boore 1999). Mitochondrial genes are separated by a total of 55 bp of intergenic spacer sequences, which are spread over eight regions and range in size from 1 to 18 bp. There are 13 overlaps with all of 49 bp and the two longest overlaps (8 bp) locate between tRNA^Trp^-tRNA^Cys^ and tRNA^Tyr^-COI separately. The overall base composition of the whole mitochondrial genome is 37.1% A, 34.5% T, 17.8% C, and 10.6% G, obvious anti-G and AT bias (71.6%), falling in the AT content of ensiferan species ranged from 64.94 to 76.9% (Zhou et al. 2017).

The initiation codons of all PCGs are typical ATN (*COII, ATP6, COIII, ND4, ND4L,* and Cytb with *ATG, ND2, COI, ATP8, ND3, ND5* with ATT; *ND6, ND1* with ATA). Seven protein genes (*ND2, COII, ATP8, ATP6, ND3, ND4L, ND6*) use TAA as the termination codons, and two genes (Cytb, *ND1*) are stopped with *TAG. COI, COIII, ND5*, and *ND4* have an incomplete stop codon T. The tRNA genes were predicted by the online software MITOS (Bernt et al. 2013), and the length ranked from 63 bp (tRNA^Arg^) to 71 bp (tRNA^Val^). All tRNAs could be folded into typical cloverleaf secondary structures, except tRNA^Ser (AGN)^, whose dihydrouridine arm formed a simple loop as in most other insects. The length of 12S rRNA and 16S rRNA are 787 bp and 1306 bp respectively, separated by tRNA^Val^. The control region of *A. emarginata* mitogenome is located at the conserved position between 12S rDNA and tRNA^Ile^-tRNA^Gln^-tRNA^Met^ gene cluster and 1407 bp in length.

Phylogenetic analyses of Meconematinae species were performed on the concatenated datasets of 13 PCGs, 22 tRNA and two rRNA genes by IQ-tree (Trifinopoulos et al. 2016). The result showed that newly sequenced species *A. emarginata* clustered with *Xizicus howardi* with high bootstrap value supporting. *A. emarginata* and *Xiphidiopsis gurneyi* were scattered within the genus *Xizicus* species clade (Figure 1).

**Figure 1.**
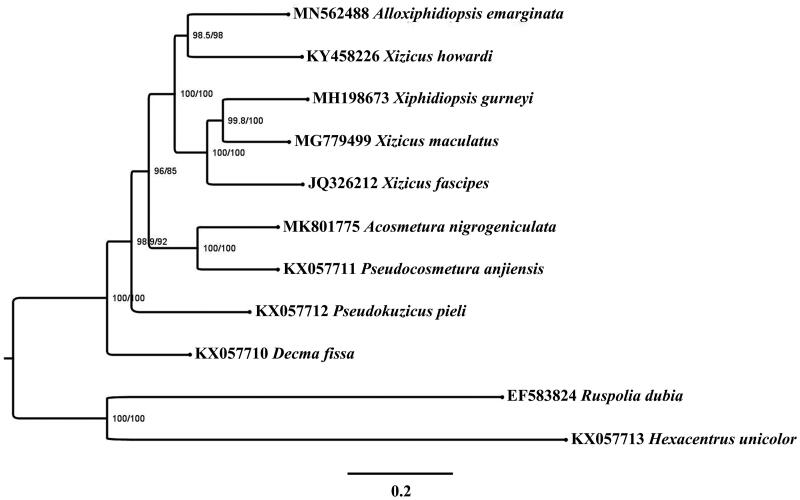
Phylogenetic reconstruction of Meconematinae using mitochondrial PCGs, tRNAs, and rRNAs concatenated dataset. Numbers in the tree represent SH-aLRT support/ultrafast bootstrap support values.
